# The road to conception for women with multiple sclerosis

**DOI:** 10.1177/20552173211032313

**Published:** 2021-07-15

**Authors:** Dessa Sadovnick, Maria Criscuoli, Irene Yee, Robert Carruthers, Alice Schabas, Penelope Smyth

**Affiliations:** Department of Medical Genetics, University of British Columbia, Vancouver, BC, Canada; Faculty of Medicine, Division of Neurology, University of British Columbia, Vancouver, BC, Canada; Department of Medical Genetics, University of British Columbia, Vancouver, BC, Canada; Faculty of Medicine, Division of Neurology, University of British Columbia, Vancouver, BC, Canada; Department of Medicine (Neurology), University of Alberta, Edmonton, Alberta, Canada

**Keywords:** Multiple sclerosis, pregnancy, conception, disease modifying therapies, family planning

## Abstract

**Objective:**

The objective of this prospective “real world” study is to gain insight into the different “roads to conception” that women with MS take as part of the prospective Canadian Multiple Sclerosis Pregnancy Study (CANPREG-MS).

**Methods:**

Participants are women with MS who are planning a pregnancy. Data cut-off for analyses was April 30, 2020.

**Results:**

We believe this is the first prospective National study of women with MS planning pregnancies.

The data are for the first 44 women enrolled of whom 26 achieved pregnancy by cut-off date. Seven women used assisted reproductive technologies (ARTs); 6 stopped disease modifying therapy (DMT) against their neurologists’ recommendations; 6 had an interruption(s) in trying to conceive due to MS relapses, MRI-detected inflammation, or limited “windows of opportunity” between DMT courses.

**Conclusion:**

The study illustrates the roads that women take to conception, even if they are on the same therapy and have similar clinical expression of MS. Advice given by treating neurologists on washout periods show discrepancies. This paper highlights the real problem that there is no definitive, international consensus on managing these women due to the lack of “real world” data and thus the goal of CANPREG-MS is to provide such real world data.

## Introduction

Multiple sclerosis (MS) preferentially affects women with the clinical onset usually occurring during the reproductive years. Historically, women with MS were advised against pregnancy. The current situation with respect to disease modifying therapies (DMTs)^1^ and revised diagnostic criteria^2^ have led to diagnosis earlier in the disease course. Thus, it appears that the number of women considering reproduction has increased in many geographic regions.^3^ Family planning has now become a major topic of interest to the MS community.^4,5^

There is a recognized need for population-based MS-specific pregnancy registries^6,7^ and this is the goal of the “Canadian Multiple Sclerosis Pregnancy Study (CANPREG-MS)”.^8^

Ideally women with MS should plan pregnancies^9,10^ but the “road to conception” can be daunting and has not been systematically prospectively studied in a “real world” scenario. Most women planning a pregnancy seek information from various sources including their neurologists, MS nurses, and social media. There are no definitive guidelines for DMT safety during pregnancy. Product information, United States Food and Drug Administration (FDA) postings, pharma pregnancy registries and committee guidelines can be outdated, confusing and/or contradictory.^11–21^ The current consensus in many regions is that for most DMTs, continuation during conception and gestation is permissible if the benefits to the mother outweigh the risks to the fetus.^9,10^ Final decisions made by woman may or may not concur with the advising source(s).

Here we present data on Canadian women with MS who were identified as “planning a pregnancy” as part of CANPREG-MS.^8^ The data are preliminary as the study is ongoing and sample size will increase. Nevertheless, we feel that this sample of 44 women provides important real world information on challenges faced by women with MS in making reproductive decisions.

## Methodology

CANPREG-MS rationale and methodology have been detailed elsewhere.^8^ Ethics approval was given by the University of British Columbia Clinical Research Ethics Board and Vancouver Coastal Health Research Institute.

CANPREG-MS was designed to include women with MS who were either pregnant or planning a pregnancy at the time of enrollment. This paper focuses on women planning to conceive. Upon conception, the woman is transferred to the “pregnancy” arm of CANPREG-MS.

Participants are followed longitudinally. Data is collected through telephone interviews using standardized questionnaires. A secure web-based application REDCap (Research Electronic Data Capture) designed exclusively to support data capture for research studies was used to develop data entry forms.^22^ Each woman’s self-reported information on MS history (MS course, DMT, etc.) has been confirmed by her neurologist after she signed a “release of information” form.^8^

Data presented here have a cut-off date of April 30, 2020. All results are truncated for this date as given the size of the dataset, it is impossible to conduct analyses without a firm cut-off.

## Results

Forty-four women planning a pregnancy were consented and enrolled. The average age at the initial interview was 31.91 years (SD = 2.96). Thirty-nine of the 44 women (88.64%) had European ancestry. Thirty of the 44 women (68.18%) had post-secondary education, 40/44 (90.91%) were employed and 42/44 (95.45%) were in a stable relationship (see Table 1).

**Table 1.** Demographics of women with MS with “planning pregnancy” status at initial interview.

Variable		N = 44	
Age at initial interview (years)		Average = 31.91	SD = 2.96
		N	%
Maternal ethnicity	Paternal ethnicity
Asian	Asian	2	4.55
European	European	30	68.18
European	European/partial information	3	6.82
European	Asian	2	4.55
European	Unknown	1	2.27
European/partial information	European	1	2.27
European/partial first nations	European	1	2.27
European/partial first nations	Unknown	1	2.27
Caribbean	Caribbean	1	2.27
Middle-Eastern	Middle-Eastern	1	2.27
No information	No information	1	2.27
Education level	
High school		1	2.27
High school & 1–2 years of college courses		3	6.82
College certificate or diploma		10	22.73
Bachelor’s degree		21	47.73
Master’s degree		6	13.64
Medical degree		2	4.55
Earned doctorate		1	2.27
Occupation	
Working at various jobs or workplaces (employed)		40	90.90
Stop working due to MS		2	4.55
Stay-at-home mother		2	4.55
Marital status	
Married		35	79.55
Common-law/long-term relationship		7	15.91
Not in a relationship		2	4.55
Previous pregnancy	
None		22	50.00
One		15	34.09
Two or more		7	15.91

The average age of MS onset was 26.05 years (SD= 5.39). The average age of diagnosis was 27.20 years (SD= 4.34) with 29/44 (65.91%) diagnosed within a year of clinical onset. The majority experienced sensory or visual symptoms at onset. See Table 2 for clinical characteristics of this cohort.

**Table 2.** Clinical characteristics of women with MS with “planning pregnancy” status at initial interview.

Variable	N = 44		
Age of MS onset (years)	Average = 26.05		SD = 5.39
Age of diagnosis (years)	Average = 27.20		SD = 4.34
Time from onset of MS to diagnosis	N		%
Within a year	29		65.91
1–2 years	7		15.91
Over 2 years	8		18.18
MS duration (MS onset to initial interview in years)	Average = 5.86		SD = 5.05
Time between initial interview & conception attempt (years)^a^	N = 33		SD = 0.41
	Average = 0.52		
Self-reported time trying Plus Time between initial interview & conception attempt (years)^b^	N = 37		
	Average = 0.87		SD = 1.01
Number of initial symptoms	N		%
One	27		61.36
Two	14		31.82
Three or more	3		6.82
Number of comorbid diseases	N	%	
None	8	18.18	
One	15	34.09	
Two	11	25.00	
Three	6	13.64	
Four or more	4	9.09	

^a^11 participants only had Initial Interview at the cut-off date.

^b^7 participants only started trying.

Reported comorbidities in these women are shown in Table 3. Few, other than reproductive conditions, affect the ability to conceive.

**Table 3.** Comorbid diseases reported by the 44 participants.

Comorbid diseases	N (Total = 44)	%
Anxiety or panic disorder	11	25.00
Depression	10	22.73
Postpartum depression (PPD) in prior pregnancy	1	2.27
Other mental health issues*(Attention deficit hyperactivity disorder; Personality disorder; post-traumatic stress disorder)*	3	6.82
Atopic dermatitis (Eczema)	1	2.27
Crohn's disease	1	2.27
Inflammatory bowel disease (IBS)	4	9.09
Grave's disease	1	2.27
Hashimoto's disease	1	2.27
Endometriosis/other uterine pathology	1	2.27
Ovarian cysts or uterine fibroids	8	18.18
Infertility	4	9.09
Polycystic ovarian syndrome (PCOS)	2	4.55
Other reproductive conditions *(Cervical cancer; HPV-precancerous lesions; Dysmenorrhea; Nabothian cysts; Uterine polyps)*	5	11.36
Pelvic inflammatory disease (PID)	1	2.27
Pemphigus vulgaris	1	2.27
Pernicious anemia	2	4.55
Psoriasis	1	2.27
Thyroid disease	5	11.36
Cancer (including skin cancers)	2	4.55
Other chronic conditions *(Shingle in the past; Prone to blood clot; Acne; Plantar fasciitis & patellofemoral disorder; Iron deficiency in the past; Pernicious anemia in the past; Hypertension)*	7	15.91

Physician correspondence relevant to CANPREG-MS^8^ tend not to give recent EDSS.^23^ However, at each study interview, Patient Determined Disease Steps (PDDS) validated for MS are scored.^2,24,25^ As done by others, (e.g. see^24,26^) PDDS scores were classified into 3 distinctive disability groups as follows: no or mild (PDDS 0-1), moderate (PDDS 2-3) and severe (PDDS 4 or higher).

Thirty-seven of the 44 (84.09%) had a consistent “no” or “mild” disability (PDDS= 0 or 1) from the initial interview until the last interview before the cut-off. Seven entered the study with moderate disability (5 had a PDDS = 2; 2 had PDDS = 3). No woman had a PDDS score ≥ 4 on enrollment.

## DMT usage

DMTs are discussed here using generic names as well as the trade name to reflect the information conveyed by participants. Physician information provided to the study used trade names, generic names, or both. We did not find any discrepancies between the patient information and that of the neurologist with respect to any specific DMT usage.

DMTs can be administered as injections, oral medications, or infusions.

Participant DMT treatment choice was often influenced by how the DMT is administered.

See Table 4 for the number of women on each DMT (or naïve to therapy) and PDDS at enrollment, pregnancy status by cut-off date and the use of assisted reproductive technology (ART). A total of 7 women used ART.

**Table 4.** Number of women on each disease modifying therapy (or therapy naïve) and PDDS at initial interview (Total = 44), status by cut-off date of April 30, 2020, and the use of ART.

Therapy: generic & trade name^a^	Number of women	PDDS at enrollment^b^	Achieved pregnancy	Still trying	Stopped trying
Treatment naive	6	5 no or mild; 1 moderate	3^c^	2	1^c^
Dimethyl fumarate (Tecfidera)	10^d^	9 no or mild; 1 moderate	5	3	1
Glatiramer acetate (Copaxone)	6	6 no or mild	3	2^c^	1^c^
Ocrelizumab (Ocrevus)	5	3 no or mild; 2 moderate	4	1	0
Alemtuzumab (Lemtrada)	5	4 no or mild; 1 moderate	3	1	1^c^
Fingolimod (Gilenya)	3	3 no or mild	2^c^	1	0
Natalizumab (Tysabri)	2	1no or mild; 1 moderate	1	1^c^	0
Interferon beta 1-a (Rebif)	2	2 no or mild	2	0	0
Cladribine (Mavenclad)	2	2 no or mild	1	1	0
Interferon beta 1-a (Avonex)	1	1 moderate	0	1	0
Teriflunomide (Aubagio)	1	1 no or mild	1	0	0
Synthetic glatiramer acetate (Glatect)	1	1 no or mild	0	1	0

^a^Neurologist records and patient reports vary in using trade and generic names. This also provides clarity if there is more than 1 trade name for a generic (e.g. interferon beta 1-a).

^b^Disability groups: No or mild – PDDS= 0,1; Moderate – PDDS= 2,3 (see literature^24,25^).

^c^Number includes 1 woman who used assisted reproductive technology (ART).

^d^Includes one woman lost to follow-up before cut-off date.

To truly reflect the information as collected, we decided to show results for DMT naïve women first followed by those who have used DMTs prior to or during the period when they were planning to conceive. DMTs are listed by the number of study participants on each (see Table 4).

### DMT naïve

Six participants were DMT naïve at enrollment. Three were recently diagnosed and wanted to conceive as soon as possible. Over time, one newly diagnosed woman stopped trying to conceive because of MS severity and began her first DMT (ocrelizumab). At cut-off, 2 woman are still trying to conceive and 3 were able to conceive.

### Dimethyl fumarate (Tecfidera; oral)

At study enrollment, 10 women were either on a washout or actively trying to conceive. Washout periods suggested by their neurologists ranged from 2 months to 12 months. At enrollment, one woman made the decision to remain on dimethyl fumarate until conception, i.e., no washout. She is currently pregnant and only discontinued dimethyl fumarate once the pregnancy was confirmed. As of the study cut-off date, no concerns have been raised by prenatal testing. One woman had planned a 3-month washout but her MS became aggressive. She stopped trying to conceive and resumed the DMT. Seven women have had no or mild disability (PDDS= 0,1) despite having stopped the DMT. Four of the 7 women achieved pregnancy including one early miscarriage before cut-off. One woman had moderate disability (PDDS = 2) which has remained stable despite her being off DMT as she is still trying to conceive.

### Glatiramer acetate (Copaxone; injectable)

Six women were on glatiramer acetate and all had no or mild disability (PDDS = 0 or 1). Three have achieved pregnancy (one had an early miscarriage and 2 are still pregnant) by the cut-off date; 2 are still trying to conceive; and one has stopped trying due to fertility issues. No washouts were recommended by neurologists but 2 women decided on their own to take the DMT “sporadically” while trying to conceive.

### Ocrelizumab (Ocrevus; infused)

Five women were on ocrelizumab at or prior to enrollment. Initial PDDS for these women were 0 (N = 2), 1 (N = 1), 2 (N = 1) and 3 (N = 1). Washout periods varied. Washouts of 2 and 3 months were recommended to 2 women. One achieved pregnancy but had an early miscarriage; and the other is pregnant at cut-off. No information on the fetus is available at this time.

One woman became pregnant 6 weeks after her second dose of ocrelizumab. Another woman is trying to conceive after her first dose. The final woman in this cohort had a six-month washout followed by 2 months of intense trying to conceive before having a second dose of ocrelizumab. At cut-off, she is pregnant, having conceived 8 months after her last dose.

### Alemtuzumab (Lemtrada; infused)

Five women were on alemtuzumab. Four had no or mild disability (PDDS 0,1) and one had moderate disability (PDDS = 2). All had the required washout and 3 became pregnant by cut-off. One woman is still trying to conceive. One woman was diagnosed as “infertile” based on her inability to conceive but no etiology was known. She and her partner decided to adopt rather than undergo in vitro fertilization (IVF) as suggested by her specialist.

### Fingolimod (Gilenya; oral)

Three women, all with no disability (PDDS = 0), were on fingolimod. One had a 2-month washout and the other a 3-month washout, as suggested by their neurologists. The third woman had stopped fingolimod and was about to start ocrelizumab when she decided it was time to try to conceive. To date, she is off any DMT. No woman had a rebound relapse after stopping fingolimod.

### Natalizumab (Tysabri; infused)

Two women were on natalizumab (one initially with no or mild disability (PDDS = 0 or 1) and one with moderate disability (PDDS = 2). They reduced the frequency of natalizumab while trying to conceive (to 6 weeks from 4 week intervals). At cut-off, one is still trying to conceive, and her disability has progressed from mild (PDDS = 1) to moderate (PDDS = 2). The other woman did not take natalizumab once pregnancy was confirmed despite her neurologist suggested a dose before delivery. She resumed the DMT at 1-month postpartum.

### Interferon beta 1-a (Rebif; injectable)

Two women in this study, both with no or mild disability (PDDS = 0 or 1), were on interferon beta 1-a. One woman decided on a 1-month washout (did not speak with her neurologist), eventually became pregnant and stayed off DMT. The other woman stayed on the DMT while trying to conceive on the advice of her neurologist. She subsequently became pregnant and had an early miscarriage. She continued to participate in CANPREG-MS and did not take any DMT during her second attempt to conceive. She is now pregnant.

### Cladribine (Mavenclad; oral)

Two women with no or mild disability (PDDS = 0,1) were on oral cladribine. One had a single course followed by an 11-month washout and she became pregnant at cut-off. The other had a 6-month washout after her second course before trying to conceive and is still trying.

### Interferon beta 1-a (Avonex; injectable)

One woman was on interferon beta 1-a (Avonex) and no washout was planned. She is still on the DMT and trying to conceive.

### Teriflunomide (Aubagio; oral)

One woman on teriflunomide did an accelerated (4 month) washout using activated charcoal^27^ and is pregnant at the cut-off date.

### Synthetic glatiramer acetate (Glatect; injectable)

One woman was on this (PDDS = 1) and is continuing her therapy while trying to conceive. She has no plans for a washout or discontinuation if she conceives.

## Discussion

CANPREG-MS is the first prospective, real world study on women with MS who are actively planning a pregnancy. Women use various sources for information about the safety of DMT usage at conception. Even with the relatively small numbers presented here, we found that 6 women discontinued their DMTs while trying to conceive against the advice of their neurologists because of disease activity. Another 6 women interrupted/stopped trying to conceive because of a major clinical relapse, a MRI-detected inflammation or limited “windows of opportunity” between DMT courses.

The data presented here are preliminary, but they show how complex the road to conception can be for women with MS. Further potentially complicating the path to pregnancy is the fact that there is no single authority or roadmap on what to do for any woman with MS with respect to therapy and disease course if she wishes to conceive. It is imperative to remind health care professionals (most often MS neurologists or MS nurses) that one must always ask female patients about reproductive plans at each visit (until pregnancy is no longer an option). This must be done regardless of whether or not a therapy is changed. Assumptions about reproductive plans based on facts presumably known to the health care professional such as age, relationship status and MS disability cannot be made. Decisions can be very surprising (e.g. a wish to be a surrogate, MS diagnosis not disclosed to partners, etc.).

Given the small sample, it is not possible to draw definitive conclusions on fertility. However, the percentage of our participants who used ARTs is similar to reports for the general Canadian population.^28,29^ It is important to consider that counseling should be given about the possibility of an increased risk for MS relapse after ART.^29,30^

As with most research, CANPREG-MS has benefits and limitations. As previously stated, this is a real world scenario with no restrictions on a woman’s age, disability, MS duration, therapy, etc. There are however certain caveats to be recognized in addition to the fact that the results are preliminary. Numbers will increase as CANPREG-MS continues. All participants were actively trying to conceive; no unplanned pregnancies were included. No participant had a PDDS of 4 or higher. European ancestry predominated. Most women (42/44) were in a stable relationship and were relatively highly educated. The Canadian healthcare system supposedly provides equal access but this is not the case regarding DMTs. Access to MS neurologist and nurses, especially in some remote communities, remains problematic in Canada.^31^

Nevertheless, as the data are presented with sufficient demographics, the study population is well defined and thus can be applied to the general comparable MS populations internationally.
